# Exploring the catalytic hydrothermal liquefaction of Namibian encroacher bush

**DOI:** 10.1038/s41598-024-83881-8

**Published:** 2025-01-02

**Authors:** Luis Cutz, Nikos Bias, Majd Al-Naji, Wiebren de Jong

**Affiliations:** 1 Process and Energy Department, University of Technology of Delft, Leeghwaterstraat 39, 2628 CB Delft, The Netherlands; 2 BasCat-UniCat BASF JointLab, Technische Universität Berlin, Hardenbergstraße 36, Sekr. EW K-01, 10623 Berlin, Germany

**Keywords:** HTL, Biomass, Invasive species, Biochar, Catalyst, Biofuel, Biofuels, Solid biofuels

## Abstract

An urgent ecological issue is the threat posed by invasive species, which are becoming more widespread especially in Africa. These encroachments damage ecosystems, pose a threat to biodiversity, and outcompete local plants and animals. This article focuses on converting *Acacia Mellifera* from Namibia, commonly known as encroacher bush (EB) into high-quality drop-in intermediates for the chemical and transport industry via hydrothermal liquefaction (HTL). HTL tackles the growing need for sustainable energy carriers while simultaneously halting the spread of the invasive species. A surface response methodology was used to optimize the HTL process for the following operational conditions: temperature (250–340 °C), residence time (5–60 min) and catalyst loading (0–10 wt%). The catalyst of choice was determined after evaluating the energy recovery (ER) of four different catalysts (Zeolite, La_2_O_3_, Hydrotalcite, Ni/SiO_2_–Al_2_O_3_) under the same HTL operational conditions. The results indicate that the addition of hydrotalcite results in high yields of bio-crude oil (13–28 wt%), without compromising the high heating value (HHV, 26–31 MJ/kg), water content (0.47 wt%) or increasing the content of oxygenated compounds compared to the non-catalytic experiment. For the experimental conditions tested, we observed a global maximum in conversion in the 330 °C and 30 min range. Our findings indicate that the most significant factor on the conversion of EB into bio-crude oil was temperature, followed by the catalyst loading. Furthermore, biochars produced at 330 °C and 30 min show potential as solid biofuels with HHVs up to 28.30 MJ/kg.

## Introduction

Concerns about climate change and anthropogenic CO_2_ emissions have prompted regulations and investments in new fossil fuel alternatives. Fossil fuels are widely used in all sectors (industrial, transportation, commercial, and residential), and only technology integrated in robust supply chains and the global energy infrastructure will be capable of reorienting to greener routes in a reasonable timeframe^1^. Meeting this target will require to find options that are not resource intensive, with less reliance of critical raw materials, do not compete with land and land-based resources such as low-risk feedstocks for bioenergy. A very promising low-risk feedstock lies within the heart of Namibia. An abundant and readily available second-generation biomass feedstock commonly known as “Encroacher Bush” (EB), *Acacia Mellifera*.

Namibia is currently facing an environmental crisis with the phenomenon of bush encroachment, due to its rapid spread, threat to biodiversity and groundwater depletion^2^. It is estimated that more than 45 million hectares of Namibia’s land are affected by it^3^. The continuous thickening of EB makes this land a threat to the national economy but offers an opportunity for biomass production. Recent estimates indicate that the available biomass from EB is around 260–300 million tonnes per year^4,5^. Furthermore, Acacia Mellifera trees and shrubs are known to exhibit rapid regrowth following thinning^6^. A recent study^6^ analyzing 13 blocks (also called plot pairs) in north-central Namibia indicated that the plots that underwent thinning exhibited a 34% increase in the number of trees and bushes below 1 m in height, as compared to the non-thinned area. This observation occurred approximately 7.2 years following the thinning process.

As of now, only a small fraction of EB is used for charcoal and firewood production, while the leftovers from processing are burnt on the field^5^. Charcoal production improves the ecosystem by de-bushing, however it is not significant against bush encroachment. Thus, Namibia needs cost-effective strategies that use EB more efficiently and innovative technology for the production of high value-added products from it.

Among the different thermochemical processes to convert EB, hydrothermal liquefaction (HTL) offers an opportunity to produce “drop-in” intermediates that later on can be converted into transport fuels, chemicals or bio-products^7,8^. For example, “drop-in” intermediates from HTL can help to decarbonize the main industrial feedstock chemicals such as benzene, toluene and aromatics from fossil feedstock. HTL comprises mixing biomass with water to create a slurry (5–30 wt%/t dry biomass) and heating the mixture to 250–370 °C at moderate pressures (5–22 MPa) for a short residence time (15–60 min)^9^. The products from HTL are a bio-crude oil, biochar, aqueous phase and gas. The main advantages of HTL bio-crude oil are the low oxygen content (10–20% less) and high heating value (HHV) higher (35–40 MJ/kg) than typical pyrolysis oils^10^.

Despite HTL’s advantages, one of the more critical challenges involves increasing the bio-crude oil yield to a level where the process is cost-effective, while reducing the high number of oxygenated compounds and improving its HHV^11^. These obstacles can be lessened with the addition of heterogeneous catalysts (transition metals and their oxides, zeolites, alkaline earth metals) which are known for their high activity, thermal stability and recyclability^7,12^. However, heterogenous catalysts accumulate in HTL biochar, reducing its quality and increasing environmental concerns, especially when using transition-metal catalysts (e.g., Nickel, Manganese, Cobalt, Platinum, among others)^12^.

The objective of the present study is to experimentally confirm the feasibility of producing a high-quality bio-crude oil from EB. So far, the only Acacia species that has been investigated for HTL is *Acacia mangium*, while EB has not been the subject of any research^13^. *Acacia mangium* oil yields are among the greatest when compared to other examined waste for HTL, such as bagasse, coconut residues, oil palm residues, and rice residues^13^. These findings suggest that Acacia species have the potential to be transformed into biofuels by HTL. Furthermore, HTL offers the opportunity to handle different type of biomass, wet and dry, increasing the robustness of potential HTL plants in Namibia. Thus, valorizing Namibian EB through HTL could help to achieve a number of important Sustainable Development Goals (SDGs), such as increasing employment opportunities (SDG 1, 8), reducing health risks associated with traditional biomass use (SDG 3), promoting sustainable energy production (SDG 7), and restoring ecosystems (SDG 15).

The key novelty of this study is that we tested different type of heterogenous catalysts (e.g., zeolites, oxides) to enhance the yield and quality of EB bio-oil for the fuel and chemical industry. This study uses a Central Composite Design (CCD) with varying temperatures, residence times, and catalyst loading to identify the trade-offs between process variables and yields of each of the HTL products. To determine the viability of EB HTL products to be used as “drop-in” intermediates, the bio-crude oil and biochar underwent extensive analysis. The HTL products were characterized using techniques such as CHN analysis, bomb calorimetry, gas chromatography–mass spectrometry (GC–MS), inductively coupled plasma—optical emission spectrometry (ICP-OES), Karl Fischer (KF), Thermogravimetric analysis (TGA) and X-ray powder diffraction (XRD).

## Materials and methods

### Samples of EB

Samples of EB were obtained from Namibia and shipped to The Netherlands. Prior shipment, the samples were stored in N_2_-flushed plastic bags. For shipping purposes, EB samples were milled to a particle size between 2 and 3 cm. The average bulk density of Namibian EB species is around 0.7 g/cm^3^^14^. In the Netherlands, no further conditioning was required for EB samples prior HTL experiments.

### HTL experimental procedure

HTL experiments were carried out in a 300 mL batch autoclave reactor (Parr 4560—Mini Bench Top Reactor, Moline, U.S.) at reaction temperatures varying from 250 to 340 °C and residence times between 5 and 60 min. The reactor was loaded with a pre-weighted EB in milli-Q water slurry such that a 15% dry matter content was attained. This biomass/water ratio is reported to yield the highest bio-crude oil yields^15^. Prior heating, the reactor was purged with nitrogen to obtain an inert environment and then pressurized to 0.14 MPa. The stirring speed was set to 150 rpm. The reactor was heated by a built-in electric jacket. The starting time of the experiment was recorded after the reactor reached the desired temperature. The temperature and pressure were monitored using an online controller and data logger (Parr 4848 Reactor controller, Moline, U.S.). After the reaction time was completed, the reactor vessel was cooled down first with a warm water bath and an ice bath afterwards. The pressure inside the reactor was registered, and the gas was safely discharged. The slurry was placed into beakers for further collection and extraction.

### Product collection and extraction

To collect the HTL products, the slurry from the reactor vessel was poured to a pre-weighed beaker. The stirrer and the reactor vessel were scraped and rinsed with Dichloromethane (DCM, Sigma-Aldrich 99.8% purity) to extract as much content as possible. This phase was added to the pre-weighed beaker with the slurry. Then, the slurry was vacuum filtrated using a 2.5-μm pore size filter paper (Whatman Grade 5). DCM was used to wash the filter cake until the color changed from dark brown to pale yellow. Then, the filter cake was marked as biochar and dried in an oven (Furnace Nabertherm 30–3000 °C, Lilienthal, Germany) at 105 °C for 24 h. After drying, the biochar was weighted and stored in a desiccator for upcoming analyses.

The filtrate from vacuum filtration was subjected to a liquid–liquid extraction to separate the oil phase from the aqueous phase using DCM as solvent extraction. The DCM was later evaporated from the oil phase using a rotary evaporator and marked as bio-crude oil. The water from the aqueous phase was also removed using a rotary evaporator (Heidolph-VAP^®^ Precision, Heildoph Instruments, Schwabach, Germany). Both bio-crude oil and aqueous phases were weighted and stored in a fridge at 4 °C until further characterization. The gaseous phase was calculated by difference.

### Catalyst screening

For the screening experimental campaign, four different catalysts were used: nickel on silica alumina (Ni/SiO_2_–Al_2_O_3_), lanthanum oxide (La_2_O_3_), Hydrotalcite and ZSM-5 (zeolite type). All the studied catalysts were acquired from Sigma-Aldrich (year 2024). The Ni/SiO_2_–Al_2_O_3_ catalyst was chosen as it has already been used in HTL experiments with the same experimental setup but with different type of biomass providing high bio-crude oil yields^12,16^. This is due to Ni/SiO_2_–Al_2_O_3_ enhances hydrogenation reactions providing high concentrations of hydrocarbons and ketones in the bio-crude oil^17^ and foster H_2_ production in the gas phase by means of redox reactions^12^. The La_2_O_3_ and hydrotalcite were selected based on the findings from^12,18^, who report high bio-crude oil yields compared to other metal oxide catalysts (e.g., CeO_2_ and MnO) or heterogeneous alkaline earth metals based catalysts (e.g., MgO and MgMnO_2_). La_2_O_3_ has basic sites that can break cellulose hydrogen bonds, increases hydrolysis and cracking processes, and suppresses dehydration reactions^18^. Meanwhile, while hydrotalcite catalyst is reported to increase the bio-crude oil yield by 82% compared to experiments without catalyst^19^. Furthermore, hydrotalcite present advantages over other selected catalyst in the Namibian case because it can potentially be sourced from neighboring countries such as South Africa^20^. Finally, ZSM-5 increases the bio-crude oil yield due its acidity and shape selectivity^12^, hence its selection. Overall, five experiments were performed in duplicates with the same experimental conditions, a non-catalytic one and 4 for each type of catalyst. The HTL operational conditions were 300 °C, 15 min residence time, 15 wt% dry biomass load and 5 wt% catalyst loading. This based on results from previous studies^21,22^ who report maximum bio-crude oil production at this operational condition.

### Design of experiments (DOE)

After conducting the screening campaign, we selected the best performance catalyst in terms of energy recovery to optimize the HTL process using a Design of Experiments-Response Surface Methodology (DOE/RSM) approach. A DOE/RSM determines the significance between different factors and process parameters to determine a desired output (response)^23^. Here we use Central Composite Design (CCD), which is reported as the most suitable DOE/RSM design for optimizing a response in multi-variable process such as HTL^15,24^. The design incorporates a factorial or fractional factorial pattern with center points and includes an additional set of “star points” that enables the evaluation of curvature^25^. In this study, an CCD with 3 variables and 3 levels was used to optimize the process conditions using Design Expert 2022V.3 software based on the experimental value for the bio-crude oil yield. The 3 variables were temperature, residence time and catalyst loading, while the range of each level was set to 250–340 °C, 5–60 min, 0–10 wt%, respectively. The temperature and residence time ranges were define based on previous experimental work reported for *Acacia mangium*^26^. The DOE design produced 20 experimental points, with 8 cubic points, 6 star points, and 1 center point with 6 replicates to ensure the accuracy of the HTL experiment.

### Product analysis

#### Proximate analysis, ultimate analysis and HHV of raw EB, bio-crude oil and biochar

The CHN elemental analysis of raw COP, bio-crude oil and biochar was conducted in accordance with the ASTM D529^27^ standard. The oxygen content was determined by difference.

The proximate analysis for raw EB and biochar was performed in accordance to method TP-510-42621^28^ for moisture content (MC), TP-510-42622^28^ for ash determination and ASTM D3175-20^29^ for volatile matter (VM). Fixed carbon was determined by difference. The MC was determined by drying the sample at 105 °C overnight in a convection drying oven (Furnace Nabertherm 30–3000 °C, Lilienthal, Germany) and weighed after cooling. The ash content was determined by dry oxidation of the samples at 550 °C using a Muffle Furnace (Thermo Scientific™ F6030CM-33-AVL, Waltham, U.S.). The volatile matter was obtained with a thermogravimetric analyzer (TGA SDT-Q600, Lindon, U.S.). The HHV of the raw EB, bio-crude oil and biochar was determined using a Bomb Calorimeter (Parr 6772, Moline, U.S.). All measurements for the proximate analysis, ultimate analysis and HHV were carried out in duplicate.

#### Gas chromatography-mass spectrometry (GC–MS) of bio-crude oil

The samples for GC–MS analysis were prepared by diluting the bio-crude oil with 2-propanol (VWR Chemicals) on a 1:10 mass ratio. Then filtration followed, using a syringe 0.2 μ PTFE filter (Whatman Puradisc 13). The GC–MS was performed using an Agilent 8890 gas chromatograph (Agilent Technologies, Wilmington, U.S.) equipped with an HP-5MS Ultra Inert column from Agilent (model: USR577054H), a split-splittles liner (Agilent 5190-2295) and coupled with both mass spectrometer detector. The measurement is conducted by injecting the same sample three times, more information about the detailed method for GC–MS can be found in^30^.

#### Inductively coupled plasma optical emission spectroscopy (ICP-OES) for raw EB, biochar and aqueous phase

Raw EB, biochar and aqueous phase samples were prepared by digesting approximately 0.1 g solid and diluting it in 1:3:1 volume ratio of water, hydrochloric acid and nitric acid respectively. Data for elemental composition were acquired using a Spectro-Arcos EOP combined with Spectro Smart Analyzer Vision software (SPECTRO Analytical Instruments GmbH, Germany).

#### Karl–Fischer (KF) titration for bio-oil

The bio-oil’s water content was measured using a Karl–Fischer titration apparatus (831 KF coulometer manufactured by Metrohm, Herisau, Switzerland). 1 mL of bio-oil was introduced into the titration vessel for this purpose. We recorded the mass of the sample being injected and the value of the water content once we reached the endpoint.

#### X-ray diffraction (XRD) of raw EB and biochar

XRD measurements were performed with a Bruker (Billerica, U.S.) D8 Advance diffractometer Bragg–Brentano geometry and Lynxeye position sensitive detector with a radiation source of Cu Kα.

## Results

### Effect of catalysts on the energy recovery (ER) of HTL products

**Fig. 1:**
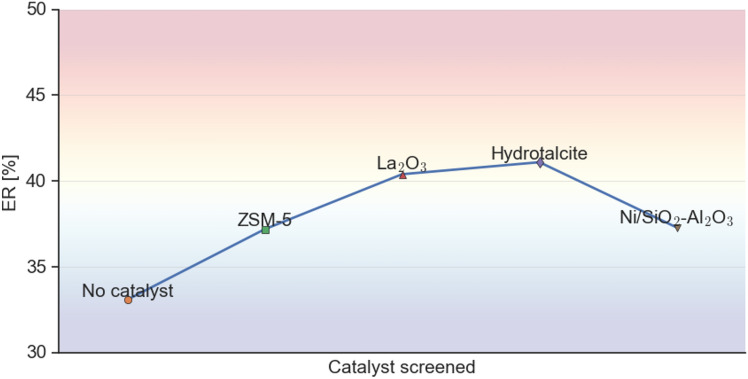
Energy recovery for EB HTL for the different type of catalysts at 300 °C and 15 min residence time.

Figure 1 summarizes the results from HTL of EB for the different type of catalysts at 300 °C and 15 min residence time. The ER indicates the percentage of the raw biomass’ energy content within the bio-crude oil. The proximate and elemental analysis of *Acacia Mellifera* is included in Supplementary Material Table S1–S2. Detailed product distribution of the HTL process, ultimate analysis, proximate analysis and HHVs of the bio-crude oil for the different catalysts is provided in the Supplementary Material Table S4–S6.

As shown in Fig. 1, all catalysts resulted an increase in the bio-crude yield, in comparison with the non-catalytic sample. The highest ER is obtained for hydrotalcite, reaching a value of 41%, while La_2_O_3_ followed with 40.4%. This phenomenon is linked to the alkaline cation and carbonate in hydrotalcite which polarize the ethereal bond (C–O–R) of EB, shifting the reaction to ionic cleavage and decreasing the production of biochar while enhancing the yield and quality of the bio-crude oil^31^. In addition, these findings are highly significant because despite literature highlights the inability of hydrotalcite to remove oxygen from phenolic compounds^12^, the EB bio-crude yield and HHV were not significantly affected when compared to the performance of the other studied catalysts. With respect to the La_2_O_3_ bio-crude oil, this contains the highest C content (65.3 wt%) and lowest O content (23.6 wt%) compared to the rest of the studied bio-crude oils (Supplementary Table S4). According to our findings, La_2_O_3_ enhanced hydrolysis and cracking reactions, which improved the quality of the HTL bio-crude oil and supported the findings reported in^12,18^. Nonetheless, lanthanum is a rare earth metal that is used mostly in the petroleum industry and battery production^32^. Its extraction is expensive and it is found naturally in rare-earth minerals such as Monazite and Bastnäsite, that are located in the USA, Brazil, India, Sri Lanka and Australia^33^. If La_2_O_3_ was to be used in a scaled-up hydrothermal liquefaction plant in Namibia, logistical challenges could arise compromising the overall sustainability of the process. Also, the accumulation of La_2_O_3_ on the biochar could potentially limit its further use and make it hazardous for applications such as soil amendment.

The lowest energy recovery was obtained for Ni/SiO_2_–Al_2_O_3_ bio-crude oil. This is consistent with literature^16^ that the use of the Ni/SiO_2_–Al_2_O_3_ catalyst gives rise to a set of trade-offs between the production of bio-crude oil and its energy content. Ni/SiO_2_–Al_2_O_3_ increased the bio-crude yield by 54% but decreased the HHV by 27% compared to the absence of catalyst. The decrease in HHV is backed up by results from the ultimate analysis (Supplementary Table S4), where the Ni/SiO_2_–Al_2_O_3_ bio-crude oil had the lowest amount of C (50.0 wt%) and the highest O (43.2 wt%) content compared to the rest of the samples. This is attributed to hydrogenation reactions which foster the production of oxygenated compounds, which reduce the C content and HHV^17^. The N content in all the bio-crude oils was relatively low (< 1.4 wt%), with the zeolite one having the lowest at 0.9 wt% (Supplementary Table S4).

### GC–MS of bio-crude oils produced from various catalysts

**Fig. 2:**
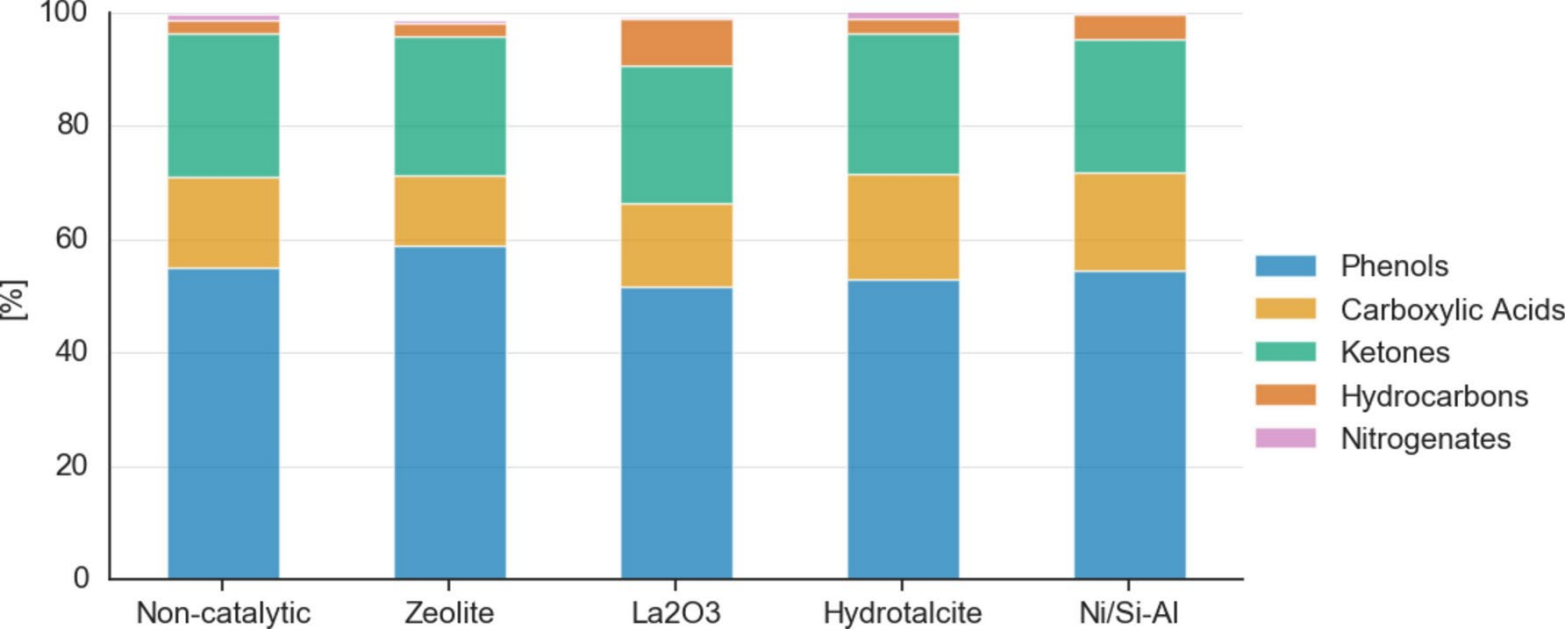
GC–MS analysis for all bio-crude oils produced via HTL for different type of catalysts at 300 °C and 15 min residence time.

Figure 2 presents the GC–MS analyses of all bio-crude oils produced from the HTL process at 300 °C for a residence time of 15 min. The detailed identification of each bio-crude oil in Fig. 2 is provided in the Supplementary Material Table S7–Table S11.

From Fig. 2 it can be observed that the bio-crude oils are highly rich in phenols. This result is in accordance with HTL literature using lignocellulosic feedstock, where catalysts promote decarboxylation reactions but are not able to hinder dehydration reactions which decompose lignin into phenolic derivatives such as 2-methoxy-phenol and 2,6-dimethoxy-phenol^19,34^. Furthermore, the 2,6-dimethoxy-phenol is typically detected in higher amounts when using water as solvent in HTL^34^. The bio-crude oil with the highest yield of phenolic compounds was the zeolite one with 59.0 wt%, while La_2_O_3_ had the lowest yield with 51.7 wt%. The high amount of phenolics in the bio-crude oil may make it an appealing raw material for bio-phenolic and epoxy resins^35^. Results from GC–MS also show that, in comparison to what is reported in literature^12^, the addition of hydrotalcite to the HTL of EB provides similar concentration of oxygenated compounds as the zeolite (ZSM-5) and Ni/SiO_2_–Al_2_O_3_. The reason behind this is because hydrotalcite facilitates oxygen removal by means of decarboxylation, but its effectiveness might be affected by the presence of butyric acid, which neutralizes the catalyst’s basic sites^19^. According to the GC–MS spectra results (Supplementary Table S10), butyric acid was not identified and thus not impacting the performance of hydrotalcite in the case of HTL of EB.

The second major type of organic compounds found in the bio-crude oils was ketones, mostly alicyclic. They are a product of the decomposition of cellulose and hemicellulose from dehydration, isomerization and aldol reactions^36^. All the bio-crude oils contained in average the same content of ketones, around 24 wt%. Carboxylic acids were also detected in the bio-crude oil, where long chain fatty acids such as oleic and n-hexadecanoic (palmitic) acid were the most prominent. Their formation is mainly attributed to the extractives contained in EB and degradation of carbohydrates^15,37^. The hydrotalcite bio-crude oil contained the highest amount of carboxylic acids with 18.5 wt%, while the zeolite one had the lowest concentration, 12.4 wt%. A bio-crude oil with high carboxylic acid content often denotes poor fuel quality because it can lead to engine and piping corrosion^18^, while it worsens stability during storage and transportation^15^. Nevertheless, depending on the post-treatment, it can be a source for the manufacturing of biodiesel^15^.

### XRD of raw EB and biochar

XRD measurements were performed on the biochar samples to evaluate the influence of HTL operational conditions on the crystallinity of the biochars compared with the parent biomass. Sharp peaks indicate higher crystallinity, while blunt peaks reveal an amorphous structure.

**Fig. 3:**
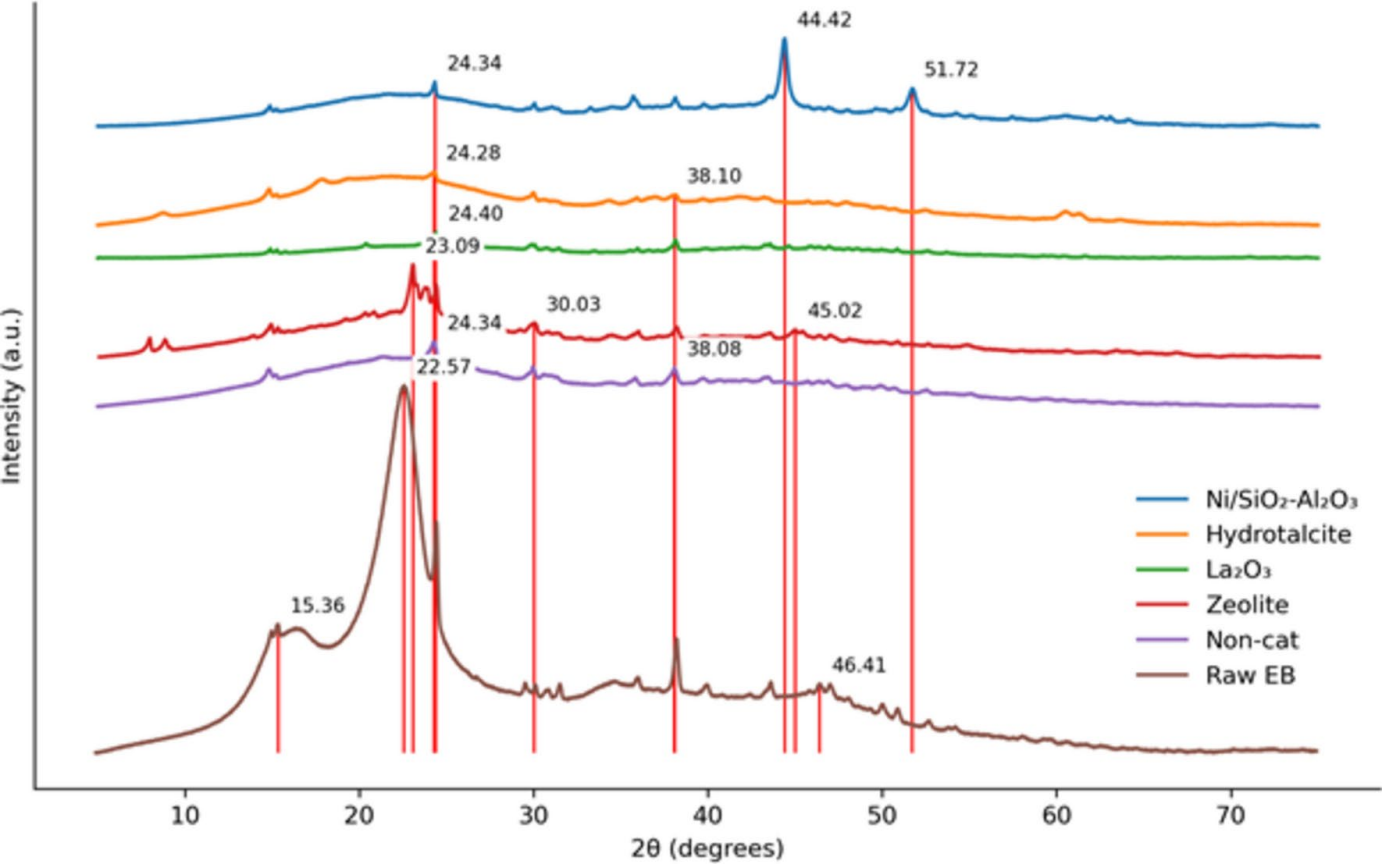
XRD pattern for raw EB and biochars obtained for various catalysts at 300 °C and 15 min residence time.

The conversion of amorphous cellulose to crystalline cellulose is observed by the flattening of the sharp peak at 2θ = 17° and 22.5° for the raw EB sample (Fig. 3). Based on the intensity of the crystalline C peak in Fig. 3, the biochars are ranked as follows: La_2_O_3_ > Ni/SiO_2_-Al_2_O_3_ > Hydrotalcite > ZSM-5 > non-catalytic. XRD results indicate that whewellite (CaC_2_O_4_·H_2_O), an stable calcium oxalate^38^, was the main crystalline mineral in all biochars at 2θ = 14.92°. However, the intensity of the whewellite peak was poor for all samples. Whewellite is considered a bio-mineral and has been detected in trees/woody species that grow on nutrient-poor soils^39^, such as EB. Further, peaks at 2θ = 8–9° and 22° indicate the presence of Sodium aluminum silicate hydrate ((Na_2_O)0.07(Al_2_O_3_)(SiO_2_)70·0.97H_2_O), which indicates the deposition of the ZSM-5 zeolite on the biochar. This is in accordance with the chemical formula of the ZSM-5 zeolite, Na_n_Al_n_Si_96-n_O_192_·16H_2_O. As for the Ni/SiO_2_–Al_2_O_3_ catalyst, the sharp peaks at 2θ = 44° and 55° confirm the accumulation of Ni on the biochar.

### Product distribution and response surfaces from CCD for HTL of EB

**Fig. 4:**
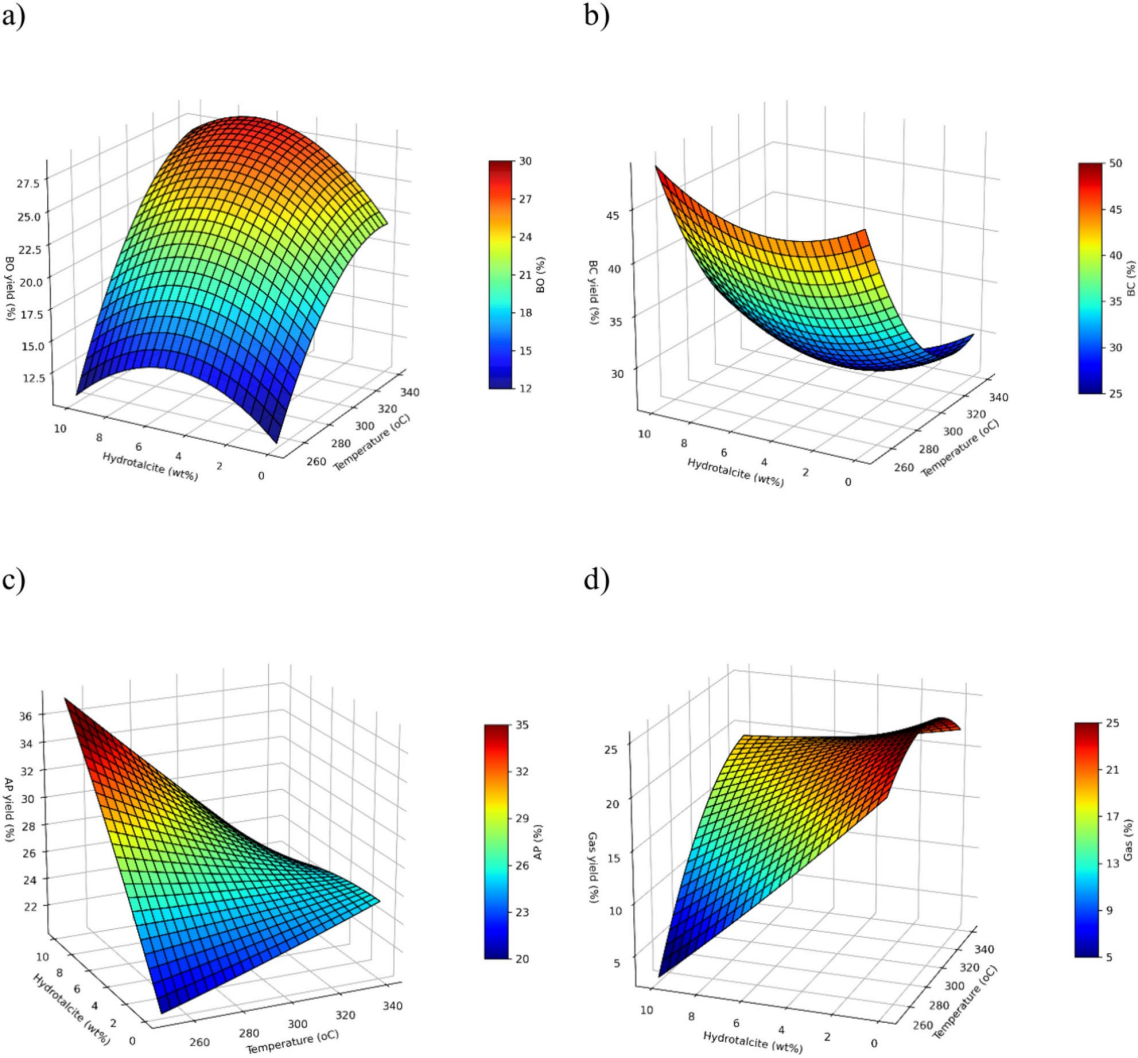
Response surfaces for each HTL product, showing effect of temperature and catalyst loading at 30 min residence time. (**a**) bio-crude (BO) oil yield. (**b**) biochar (BC) yield. (**c**) aqueous phase (AP) yield. (**d**) gas phase yield.

The response surface plots for each of the HTL products is presented on Fig. 4 as a function of the temperature and catalyst loading. This is because these variables were the most statistically significant for each predictive model (Supplementary Table S21–S24). A residence time of 30 min was chosen for better visual representation of the HTL product yields. The yields for the different products are expressed as mass yields (wt%). The fitting quadratic equations for each HTL phase are provided in the Supplementary Table S14 .

The bio-crude oil yield varied between 13.2 and 28.5% within the evaluated range of operation conditions. The highest bio-crude yield (28.5 wt%) from the 20 experiments of the CCD is obtained at 330 °C, 30 min and 7.5 wt% catalyst. The bio-crude oil model was found to be statistically significant (R^2^ = 0.88, F-value = 7.67, *p*-value = 0.0028 and RSS = 3.29). The most significant factor on the conversion of EB into bio-crude oil was temperature (*p*-value = 0.0003), followed by the catalyst loading (*p*-value = 0.0096). The residence time appears to have a less important role according to its *p*-value (0.0465). Furthermore, the benefits of catalytic HTL can also be observed from the surface response model (Fig. 4). For example, using 1.6 wt% of hydrotalcite will reduce the operational conditions (260 °C and 38 min) to reach the same yield to that of a 14.6 wt% bio-crude yield produced without a catalyst at 300 °C and 15 min. The quadratic model indicates a theoretical maximum of 29.4 wt% at higher operational conditions 340 °C, 60 min and 5% catalyst. However, our findings indicate that in average once a catalyst loading of 5% is exceeded, the production of bio-crude oil is hindered. This means that higher yields of bio-crude will not be produced at the greatest catalyst loading (10%).

The biochar model performed satisfactorily with an R^2^ = 0.89, F = 8.64, *p-*value = 0.0012 and RSS = 7.43. The surface response for the biochar yield shows an opposite trend with temperature compared to the BO one. The lower the temperature, the higher is the yield, which indicates limited devolatilization and carbonization as confirmed by proximate analysis (Supplementary Table S14 and S16). This behavior is characteristic of biochars produced via HTL^16,40^. Moreover, the addition of a catalyst shows a slight increasing trend in the biochar yield when compared to the one produced without the catalyst. This is attributed to the accumulation of the catalyst on the BC as reported in Fig. 3 and confirmed by ICP-OES results (Supplementary Fig. S3).

At higher temperatures, there is an increase in thermal degradation of the aqueous phase which breaks down the compounds into the gas phase and bio-crude oil. It is known that at high temperatures, organic compounds such as furans and furfurals breakdown and re-arrange into phenols, indoles and ketones^41,^ contributing to an increase in bio-crude yield. The aqueous phase model performed poorly with an R^2^ = 0.64, *p-*value = 0.1523, F = 1.97 and RSS = 10.18. The aqueous phase yield surface shows a significant dependence on the catalyst loading, but only at lower temperatures (< 280 °C), whereas at higher temperatures, the model indicates that neither of the studied operational variables have much of an effect on the yield. Higher amount of catalyst leads to higher aqueous phase yield. This is confirmed by ICP-OES results of the aqueous phase (Supplementary Figure S4) which show a significant average increase of Mg (18,478 ppm) and Al (83 ppm) content compared to the non-catalytic experiment.

The gas phase is highly influenced by the catalyst loading, especially at low temperatures. A high loading of hydrotalcite (10 wt%) at lower temperatures reduces the gas yield due to limited decarboxylation, which is in agreement with high biochar yields (Fig. 4b). With the increase of the temperature, all HTL products are thermally degraded reaching plateau in conversion^41^. The gas model performed satisfactorily with an R^2^ = 0.90, *p* = 0.0005, F = 10.36 and RSS = 5.34.

Finally, the bio-crude model from the CCD was optimized to maximize the bio-crude oil yield, while maintaining a HHV. Statistical analysis indicates that a quadratic model fit best the responses obtained from the 20 experimental points. The optimal operational conditions (340 °C, 60 min and 5 wt% catalyst loading) suggested by the model were validated experimentally, yielding a 26.98 ± 1.35 wt% bio-crude oil. The quadratic model overestimated the bio-crude yield with an 8.0% relative error. The error of the quadratic model is partially attributed to losses in product separation/collection and the experimental loss of some organic compounds with low boiling points during the evaporation process. In addition, the model’s inaccuracy could be attributed to two of the axial points of the CCD that are outside of the DOE cube, causing extrapolation of data over the experimental range^25^. Nonetheless, the error of the CCD model is in agreement with the relative error values (3.0–6.2%) reported by other studies^15,42^ using the DOE/RSM approach for the conversion of waste activated sludge and barley straw using HTL.

### ER of the bio-crude oils obtained from the CCD

**Fig. 5:**
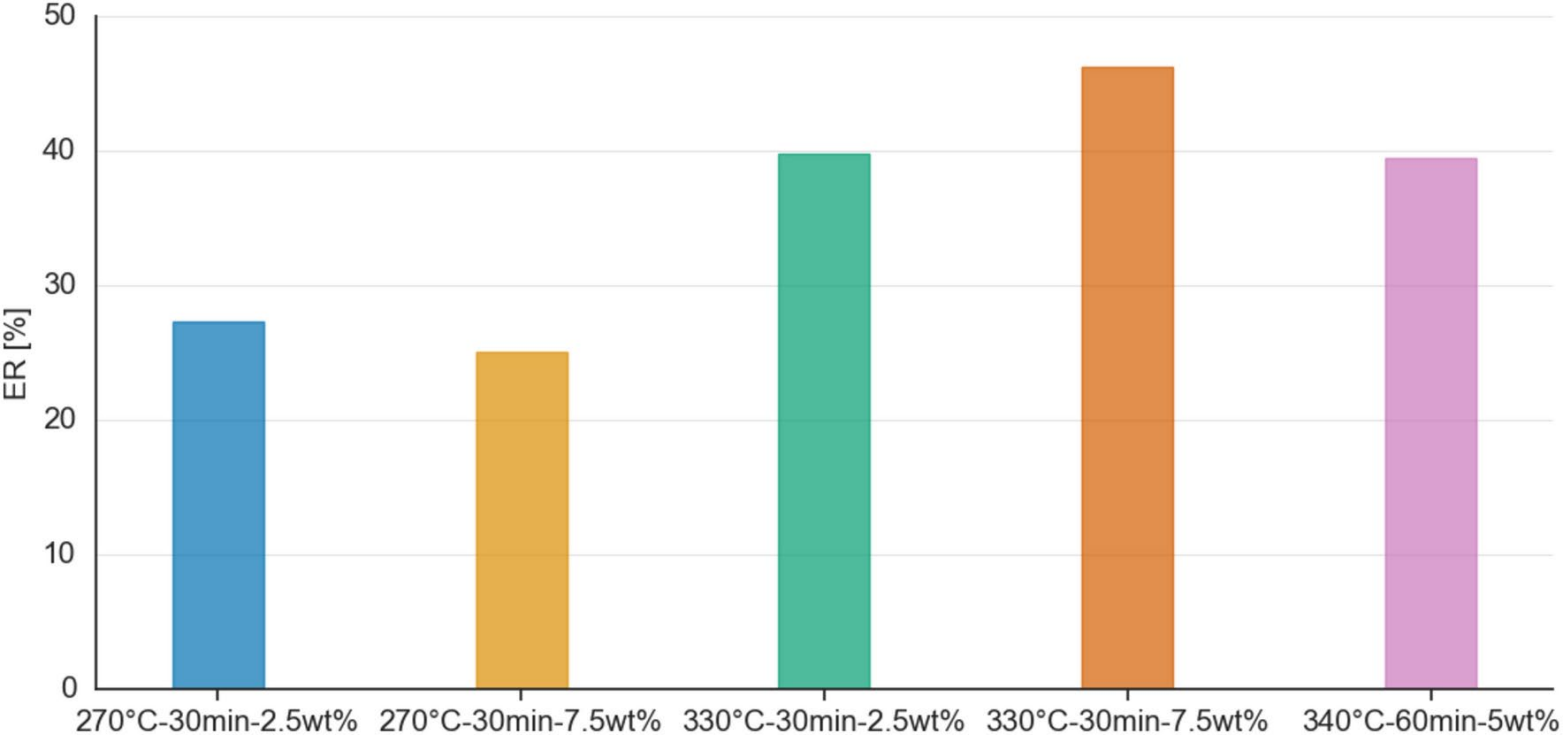
Energy recovery (ER) of the 5 EB bio-crude oil samples from the CCD.

Our study examined the impact of hydrotalcite on the ER of 5 bio-crude oils derived from the CCD approach (Fig. 5). That is, the 2 highest (330 °C–30 min–2.5 wt% and 330 °C–30 min–7.5 wt%) and 2 lowest points (270 °C–30 min–2.5 wt% and 270 °C–30 min–7.5 wt%) based on bio-crude oil yield, as well as the predicted optimal condition provided by the optimization of the CCD model (340 °C, 60 min and 5wt%).

In general, ER values for the selected points range between 25 and 46%. These results are in agreement with results reported in literature regarding HTL of lignocellulosic feedstocks, where ER ranges from 32.6 to 57.6%^19,43^. The ER values indicate that mild temperature and mild catalyst loading provided the highest ER. The bio-crude oil obtained at 330 °C–30 min–7.5 wt% has the highest ER value (46.2%), a 12.4% increase compared to the 300 °C–15 min–5 wt% point (Fig. 1). This is agreement with the ultimate analysis (Supplementary Table S15), which indicates that this sample (330 °C-30 min-7.5wt%) has a relatively high C content of 71.6% and low O content of 18.7% compared to other five oils analyzed. It is worth noting, that all 5 selected samples from the CCD have lower O content than the bio-crude oils from the catalyst screening campaign (Supplementary Table S15), except the samples produced at 270 °C–30 min–7.5 wt% and 340 °C–60 min–5 wt%. Due to this effect, these bio-crude oils (270 °C–30 min–7.5 wt% and 340 °C–60 min–5 wt%) have lower ER values compared to the two samples at 330 °C (Fig. 5). The predicted optimal condition (340 °C–60 min–5 wt%) resulted in a decrease in ER due to higher temperature and extended residence time. This may have lead to enhanced cracking of the bio-crude oil to lower energy compounds (reducing HHV) or gas formation from intermediates (reducing bio-crude oil yield)^44^. These findings are supported by results of ultimate analysis (Supplementary Table S15), which indicate that the 340 °C–60 min–5 wt% bio-crude oil has the lowest C content (61.3 wt%) and highest O content (30.3 wt%), making it the bio-crude oil with the lowest quality among the 5 studied samples. All five bio-crude oils have a water content between 0.25 and 0.47 wt% (Supplementary Fig. S2).

### GC–MS of the bio-crude oils obtained from the CCD

**Fig. 6:**
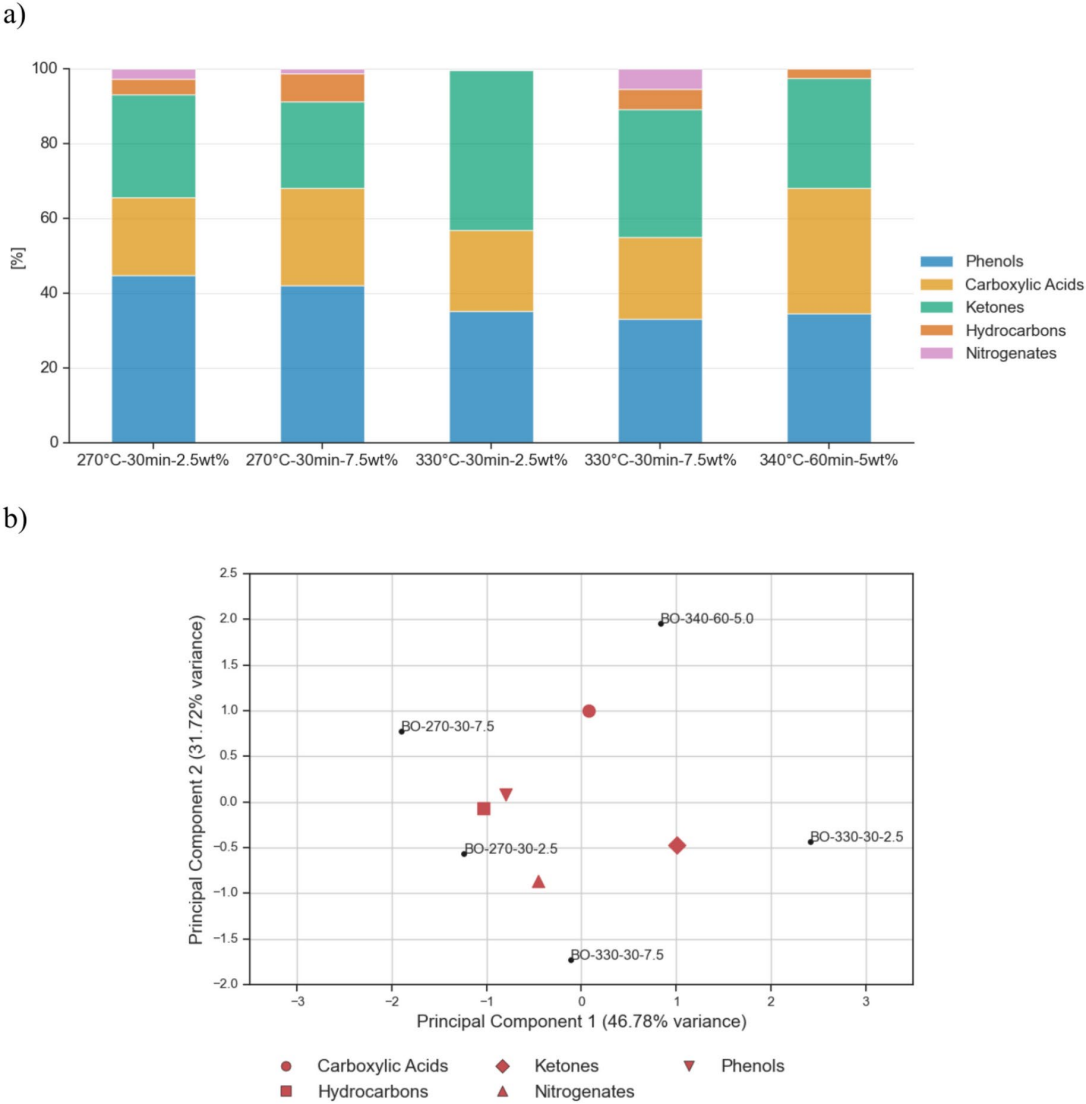
Chemical composition of the 5 EB bio-crude oils from the CCD. (**a**) GC–MS of the 5 bio-crude oils and (**b**) Principal component analysis (PCA) of GC–MS spectra of the 5 bio-crude oils.

Gas chromatography–mass spectrometry (GC–MS) has been used to examine the composition of the five bio-crude oils derived from the CCD. In Fig. 6, a significant reduction in phenolic content is observed compared to the results of the screening campaign without a catalyst and using hydrotalcite, 300 °C–15 min–5wt% (Fig. 2). In average, the concentration of phenolic compounds of the five bio-crude oils derived from the CCD is 38%, which is a 31% decrease compared to the non-catalytic experiment at 300 °C and 15 min. The maximum concentration of phenols is obtained for 270 °C–30 min–2.5 wt% (44.9%) compared to hydrotalcite’s phenol composition at 300 °C–15 min–5 wt% (53%). The bio-crude oil at 330 °C–30 min–7.5 wt% has the highest concentration of ketones and the lowest content of phenolic compounds, 34.2% and 33.2%, respectively. Furthermore, the average carboxylic acid content increased from 20.7 to 26.1 wt% compared to the 300 °C–15 min–5 wt% point of the catalyst screening campaign. These differences are attributed to the higher residence time compared to the 300 °C–15 min–5 wt%, which could have resulted in: (i) further decomposition of the phenolic compounds^45^, (ii) repolymerization of decomposed compounds on the biochar^44^, (iii) further degradation of furans or furfurals to ketones^46^ and/or (iv) further decomposition of EB’s extractives. Although previous research has shown that hydrotalcite can be used to decarboxylate microalgal pyrolysis oil^47^, GC-MS analysis of the tested EB bio-crude oils found no butyric acid formation due to the absence of esters, contradicting the hypothesis that steric hindrance from fatty acid alkyl esters can increase hydrotalcite activity.

PC1 accounted for 46.8% (Fig. 6a) of the total variance between samples and effectively distinguished between bio-crude oils at lower temperatures (270 °C) located at the most negative location on the plot, and bio-crude oils at higher temperatures (330 °C and 340 °C) positioned to the right of the plot. PC2 accounted for 31.7% of the total variance and distinguished the bio-crude oils based on their quality. Furthermore, the loadings in Fig. 6a also indicated that BO-340-60-5.0 and BO-330-302.5 have relatively greater abundance of carboxylic acids and ketones, while oils such as BO-270-30-75 have relatively greater amounts of hydrocarbons and phenols.

### CCD biochar characterization

**Fig. 7:**
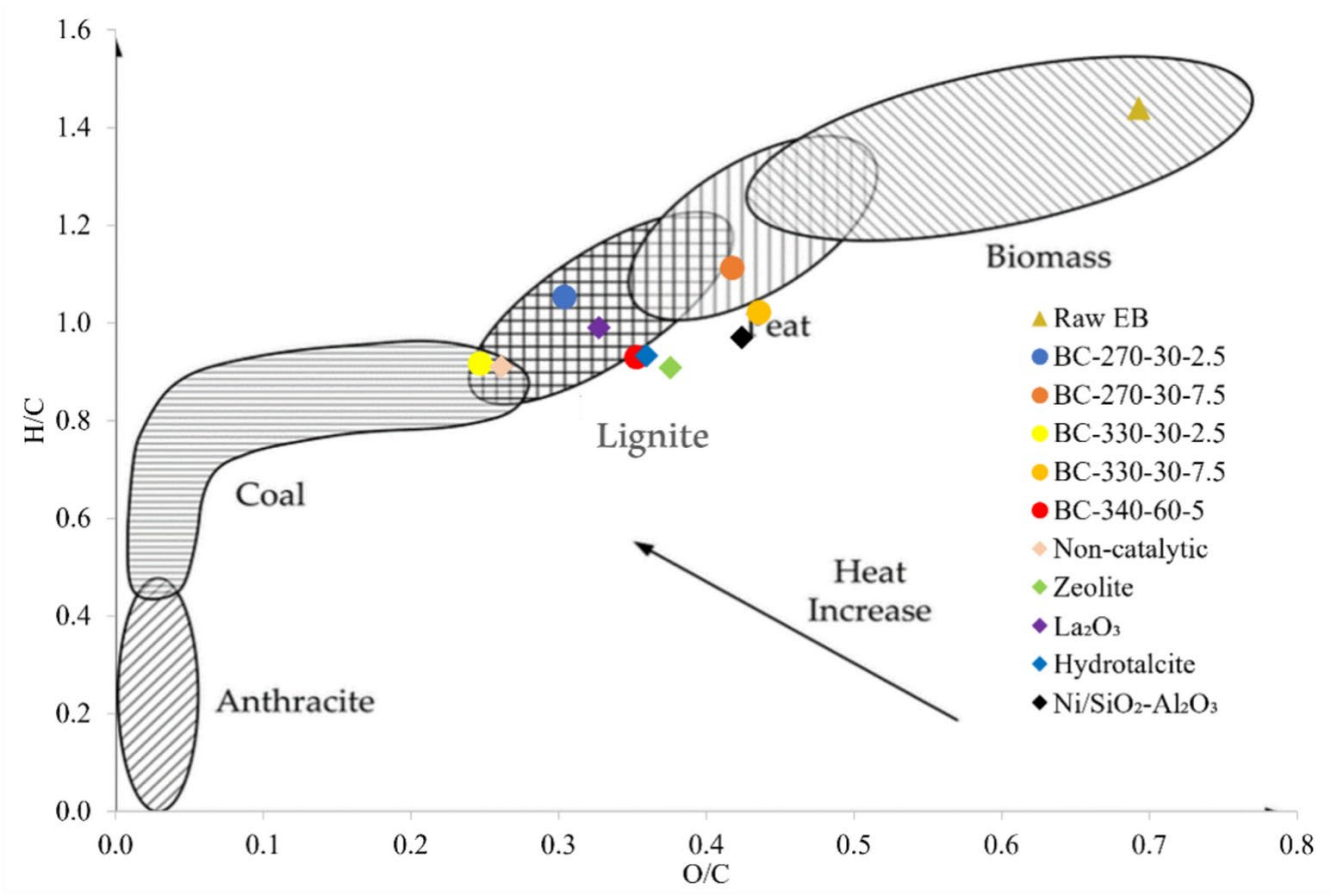
Van Krevelen diagram for the biochars produced from the catalyst screening and CCD campaign. This figure also includes a comparison with the raw EB. The biochars produced from various catalysts were produced at 300 °C and 15 min residence time.

Biofuels can be classified and compared to fossil fuels using the van Krevelen diagram^48^, which combines O/C and H/C ratios and provides a rough prediction of a fuel’s quality. In general, high ratios of H/C and low O/C are always preferred, as both high content of hydrogen and low content of oxygen lead to higher calorific values^48^. Figure 7 uses the Van Krevelen diagram to classify the raw EB, bio-crude oil and biochar produced for the catalyst screening campaign and CCD in terms of their potential as solid fuels. The H/C and O/C ratios tend to decrease as the energy efficiency of the fuel increases. Detailed ultimate analysis and proximate analysis for the biochar is provided in the Supplementary Tables S1, S4–S5, S15–S16.

According to literature^49,50^, the elemental O/C and H/C of raw EB is within the typical ranges for woody lignocellulosic biomass (Fig. 7). The proximate analysis (Supplementary Table S1) of the feedstock resembles a woody type of biomass^51^, containing high amounts of VM and very low ash content. Moisture and ash content are similar to *Acacia mellifera* species grown in other countries such as Ethiopia and Sudan, 7 wt% and 1.9–4.2 wt% respectively^52^. Further, ICP-OES analysis (Supplementary Table S2) reveals that raw EB is a “virgin” type of feedstock, as it hasn’t been polluted with metals (e.g., Ni, Cr and Cd) coming from human activity^53^, reducing the complexity of potential downstream processing.

Compared to raw EB, biochar samples have higher quality (higher C and lower O content) and can be potentially considered as a solid fuel that could replace coal or lignite. Based on the O/C and H/C ratios, the absence of catalyst favors the formation of a high-quality biochar similar to coal. Meanwhile, the biochar samples produced using catalysts fall approximately in the same graph area, which is the lignite—peat one. Thus, the use of EB biochars as solid biofuels shows promise as a viable option for generating power, especially since coal, lignite, and peat are commonly used fossil fuels for this purpose. After HTL, the quality of the CCD biochars did not show a significant improvement due the presence of hydrotalcite, except the one produced at 330 °C–30 min–2.5 wt%. The 330 °C–30 min–2.5 wt% biochar reports the best values in terms of HHV (28.30 MJ/kg) and low ash content (6.92 wt%) compared to the rest of (non-) catalytic experiments. The biochars that had a larger catalyst loading showed a decrease HHV, mostly attributed to the increased ash content. This is confirmed by the presence of alkali and alkaline earth metallic (AAEM) species in the biochars (Supplementary Fig. S3). Compared to other technologies for biochar production, the HHV values of EB biochars produced via HTL are in average 46% higher than those made through torrefaction for similar species. A study reported by^54^, indicate that torrefaction of Namibian wood (*Acacia mellifera, Acacia reficiens, Acacia erubescens, and Dichrostachys cineria*) yields HHV values between 16.98–18.90 MJ/kg. The production of high-quality biochar as a by-product of HTL shows the versatility of this process, as it yields a commodity that could be of interest to various industries.

### Practical implications and challenges

The use of EB for bioenergy is a potential solution to control its spread, ensuring the preservation of native ecosystems and biodiversity. In addition, HTL has the potential to transform the issue of EB into high-quality biofuel and biochar and provides Namibia the agency to develop local and scalable technology. These efforts contribute to decrease greenhouse emissions, modernize the Namibian energy sector and enhance energy security. EB biochar has also a wide range of applications, including its use as bio-fertilizer, in construction materials, and as solid biofuel, among other uses^57^. In addition, the development of collecting, processing, and product distribution value chains for HTL plants will positively influence the Namibian economy. This will lead to job creation and an overall improvement in the quality of life for its citizens.

When it comes to challenges, it is essential to implement advanced logistics and supply chain management systems to reduce costs and environmental impact of transporting biomass to HTL plants. Nonetheless, HTL plants can take advantage of the existing value chains of charcoal and pellets^55^. HTL’s economic viability also relies on the development of reactors capable of handling large volumes of EB, ensuring high process efficiency, upgrading the bio-crude-oil and water management^56^. This issue can be addressed by implementing modular reactor systems that can be easily scaled up or down depending on the production needs. Additionally, it is crucial to optimize the process conditions to maximize the bio-crude oil yield and quality, as shown in this work. Another important aspect is the development of methods for efficient water recycling and treatment to minimize freshwater usage in the HTL process^56^. Also, the success of HTL technology relies heavily on the access to markets for products derived from HTL. This includes ensuring that the quality of bio-oil, biochar, and other by-products meets the quality, ecological and social requirements (e.g. the Forest Stewardship Council Certification) of the various applications and contribute to the diversification of revenue streams. Forming partnerships with industry stakeholders, research institutes, and government agencies can be instrumental in the industrialization of HTL. These collaborations can provide valuable technical expertise, financial support, and opportunities to enter new markets.

## Conclusions

The potential of Namibian encroacher bush (*Acacia Mellifera*) as a feedstock for the production of high-quality drop-in intermediates was assessed using catalytic HTL. Initially, four distinct catalysts (Zeolite, La_2_O_3_, Hydrotalcite, Ni/SiO_2_–Al_2_O_3_) were evaluated under identical operating conditions. Nevertheless, hydrotalcite had the highest ER, positioning it as the most sustainable and optimal choice for evaluating the interactions of different operational variables of the HTL process using a CCD approach.

Results from the CCD approach reveals that HTL at 330 °C, 30 min, and 7.5 wt% of hydrotalcite yields the best bio-crude oil yield (28.5 wt%) without affecting HHV (31.3 MJ/kg). The use of hydrotalcite reported in average a 31% decrease in phenolic compounds content with respect to using no catalyst. All five bio-crude oils from the CCD have a water content less than 0.47 wt%. For similar species, HTL-produced EB biochars have 46% higher HHV values than torrefaction-produced biochars. The 330 °C–30 min–2.5 wt% biochar has the highest HHV (28.30 MJ/kg) and lowest ash concentration (6.92 wt%) of all (non-) catalytic experiments. Scaling up and commercialization of HTL pose significant engineering problems such as logistics, bio-oil upgrading and water management. Nonetheless, this work offers valuable insights into the reaction conditions, product yield, and characteristics needed to create high-value products from HTL. The use of hydrotalcite as a catalyst during HTL of EB was not as effective in removing oxygen from stable phenolic compounds as the other tested catalysts, but it did prevent the creation of butyric acid, which is known to neutralize hydrotalcite’s basic sites. Our findings demonstrate that the use of hydrotalcite not only enhanced the process but also allowed for a reduction in the operational conditions compared to experiments conducted without the catalyst. Some suggestions for future improvements include looking into how HTL can be mixed with other biomass sources in Namibia, evaluating the process’s life cycle, and scale-up tests in a continuous reactor. This study’s findings have far-reaching implications since they advance the HTL state-of-the-art for converting invasive species into biofuels and biochar. ecosystem. It also seeks to enhance rural economy by by identifying valuable applications for invasive species.

## Supplementary Information

Supplementary Information.

## Data Availability

Data supporting the findings presented in this paper are available within the manuscript and in the Supplementary Material.
