# Supplementary material for: Exploring the catalytic hydrothermal liquefaction of Namibian encroacher bush
Source: Sci Rep. 2025 Jan 2;15:112. doi: 10.1038/s41598-024-83881-8 (PMC11696455; doi:10.1038/s41598-024-83881-8)
Supplement: Supplementary file 1 — Supplementary Information. [file 41598_2024_83881_MOESM1_ESM.docx]

Exploring the Catalytic Hydrothermal Liquefaction of

Namibian Encroacher Bush

Luis Cutz^a,^ *, Nikos Bias^a^, Majd Al-Naji^b^, Wiebren de Jong^a^

^a^ Process and Energy Department, University of Technology of Delft, Leeghwaterstraat 39, 2628 CB Delft, The Netherlands.

^b^ BasCat – UniCat BASF JointLab, Technische Universität Berlin, Hardenbergstraße 36,

Sekr. EW K-01, Berlin 10623, Germany

# *Acacia Mellifera* (EB) data

Table S1. Proximate & ultimate analysis of *Acacia Mellifera* compared with similar feedstocks, (wt% d.b.)

| Biomass type | MC | Ash | FC | VM | HHV | C | H | O | N |
| --- | --- | --- | --- | --- | --- | --- | --- | --- | --- |
| *Acacia Mellifera* | 9.42 | 1.14 | 21.03 | 68.41 | 19.34 | 47.31 | 5.68 | 43.71 | 1.98 |
| [*Acacia Holosericea*](https://www.mdpi.com/2306-5354/6/2/33)^1^ | 9.56 | 3.91 | 21.21 | 65.32 | 18.13 | 44.03 | 5.67 | 50.05 | 0.25 |
| [*Pennisetum purpureum*](https://link.springer.com/article/10.1007/s40974-019-00139-0)^2^ | 5.93 | 7.82 | 16.81 | 69.44 | 18.55 | 46.90 | 6.29 | 45.30 | 1.27 |
| [*Acacia mangium*](https://www.mdpi.com/2071-1050/13/9/5249)^3^ | 9.98 | 2.25 | 15.38 | 72.39 | 21.26 | 48.09 | 5.66 | 45.77 | 0.48 |

Table S2. ICP-OES of raw *Acacia Mellifera*

| Element | Ca | K | Mg | P | S | Fe | Sr | Zn | Ba | B | Cu | Co | Mo |
| --- | --- | --- | --- | --- | --- | --- | --- | --- | --- | --- | --- | --- | --- |
| Concentration (mg/kg) | 11783 | 2763 | 1618 | 1045 | 854 | 124 | 48 | 23 | 21 | 17 | 8 | 3 | 3 |

The proximate analysis of EB resembles a woody type of biomass, containing high amounts of VM and very low ash content. Moisture and ash content are similar to other bush and *Acacia* species (5-10% and 1-5% respectively). Mineral and metal composition of EB shows that sulphur is present in low concentrations (854 mg/kg or ppm). The most prominent elements are Calcium, Potassium and Magnesium which belong to alkaline earth metals. Heavy metals such as Zn, Co and Mo are at very low concentrations (<30 mg/kg or ppm).

# Catalyst screening campaign data

Table S3. Product distribution (wt%) for catalyst screening campaign


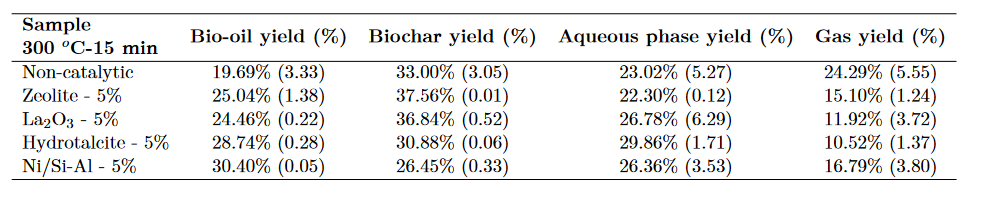


*A*ll catalysts caused an increase in bio-oil yield, in relation to the non-catalytic sample. The highest effect was achieved by the Ni/SiO₂-Al₂O₃, which yielded 30.4wt% biocrude oil, while the lowest yield was obtained when no catalyst was used, 19.7wt%. Overall, there is an increase between 24% (La₂O₃) to 54% (Ni/SiO₂-Al₂O₃) in the BO yield in comparison to not using a catalyst. Therefore, the addition of catalysts benefited the C-C cleavage of structural compounds in biomass due to the acidity (zeolite and Ni/SiO₂-Al₂O₃) or alkalinity (La₂O₃ and hydrotalcite) and porosity of these materials.

Table S4. Ultimate analysis data for catalytic screening campaign for bio-oil and biochar

| Sample | HHV (MJ/kg) | HHV STD | C (%) | H(%) | N(%) | O(%) | H/C | O/C |
| --- | --- | --- | --- | --- | --- | --- | --- | --- |
| **Bio-oil** |  |  |  |  |  |  |  |  |
| Non-catalytic | 32.54 | 0.23 | 52.7 | 6.1 | 1.3 | 39.9 | 1.39 | 0.57 |
| Zeolite | 28.76 | 0.43 | 64.5 | 9.6 | 0.9 | 25.0 | 1.79 | 0.29 |
| La₂O₃ | 31.98 | 0.19 | 65.3 | 9.9 | 1.2 | 23.6 | 1.82 | 0.27 |
| Hydrotalcite | 27.64 | 0.96 | 54.3 | 6.2 | 1.4 | 38.1 | 1.37 | 0.53 |
| Ni/SiO₂-Al₂O₃ | 23.71 | 1.23 | 50.0 | 5.7 | 1.1 | 43.2 | 1.37 | 0.65 |
| **Biochar (d.b.)** |  |  |  |  |  |  |  |  |
| Non-catalytic | 28.08 | 0.29 | 68.6 | 5.20 | 2.30 | 23.9 | 0.91 | 0.26 |
| Zeolite | 25.94 | 0.06 | 62.1 | 4.70 | 2.10 | 31.1 | 0.91 | 0.38 |
| La₂O₃ | 25.82 | 0.04 | 64.2 | 5.30 | 2.50 | 28.0 | 0.99 | 0.33 |
| Hydrotalcite | 25.68 | 0.49 | 63.0 | 4.90 | 1.90 | 30.2 | 0.93 | 0.36 |
| Ni/SiO₂-Al₂O₃ | 22.23 | 0.20 | 59.4 | 4.80 | 2.20 | 33.6 | 0.97 | 0.42 |

HHV showed an opposite trend compared to the bio-oil yield, where the highest HHV was obtained using no catalyst, 32.5 MJ/kg, while catalysts reduced it. The bio-oil yield with the lowest calorific value was the Ni/SiO₂-Al₂O₃ sample with 23.7 MJ/kg.

La₂O₃ bio-oil had the highest C content (65.3wt%) and lowest O content (23.6wt%) among the other samples. On the other hand, Ni/SiO₂-Al₂O₃ bio-oil had the lowest amount of C and the highest O content, 50.0wt% and 43.2wt%, respectively. Additionally, the bio-oil yield with La₂O₃ contained the highest amount of H, at 9.9wt%. Zeolite bio-oil yield had similar CHNO composition with the La₂O₃ sample. The N content in all the biocrudes was similar to raw EB, with the zeolite one having the lowest at 0.9wt%.

Table S5. Proximate analysis for catalyst screening campaign of biochar samples (d.b.)

| Sample | Volatile Matter (VM) (%) | Fixed Carbon (FC) | Ash (%) |
| --- | --- | --- | --- |
| Non-catalytic | 40.32 ± 0.64 | 56.11 ± 0.76 | 3.56 ± 0.12 |
| Zeolite | 35.38 ± 0.19 | 52.77 ± 1.78 | 11.85 ± 1.59 |
| La₂O₃ | 40.17 ± 0.61 | 45.88 ± 1.87 | 13.95 ± 1.27 |
| Hydrotalcite | 40.03 ± 0.49 | 52.02 ± 1.96 | 7.94 ± 1.47 |
| Ni/SiO₂-Al₂O₃ | 30.30 ± 6.34 | 44.00 ± 7.38 | 25.70 ± 1.05 |

Biochar samples from the catalytic campaign showed a significant reduction in volatile mass compared to the raw EB sample. The Ni/SiO₂-Al₂O₃ sample however showed the lowest amount of VM at 30.30%, which is caused by the high amount of ash (25.70%). The non-catalytic sample had the highest amount of fixed carbon. which is a desirable aspect when compared to other solid fuels such as coal.

Table S6. HHV and bio-oil yield fractions for ER calculation

| Sample | Non-catalytic | Zeolite | La₂O₃ | Hydrotalcite | Ni/SiO₂-Al₂O₃ |
| --- | --- | --- | --- | --- | --- |
| HHV (MJ/kg) | 32.54 | 28.76 | 31.98 | 27.64 | 23.71 |
| HHV STD (MJ/kg) | 0.23 | 0.43 | 0.19 | 0.96 | 1.23 |
| Bio-oil yield (%) | 19.69 | 25.04 | 24.46 | 28.74 | 30.4 |
| Bio-oil yield STD (%) | 3.33 | 1.38 | 0.22 | 0.28 | 0.05 |
| ER (%) | 33.1 | 37.2 | 40.4 | 41.1 | 37.3 |
| ER STD (%) | 5.6 | 2.1 | 0.4 | 1.5 | 1.9 |

From Table S6 it is observed that Hydrotalcite achieved the best energy recovery with a value of 41.0%, while La₂O₃ followed with 40.4%. The lowest energy recovery was obtained for the non-catalytic BO, at 33.1%.

Figure S1 shows the GC-MS peaks for each of the bio-oil samples from the catalyst screening campaign.


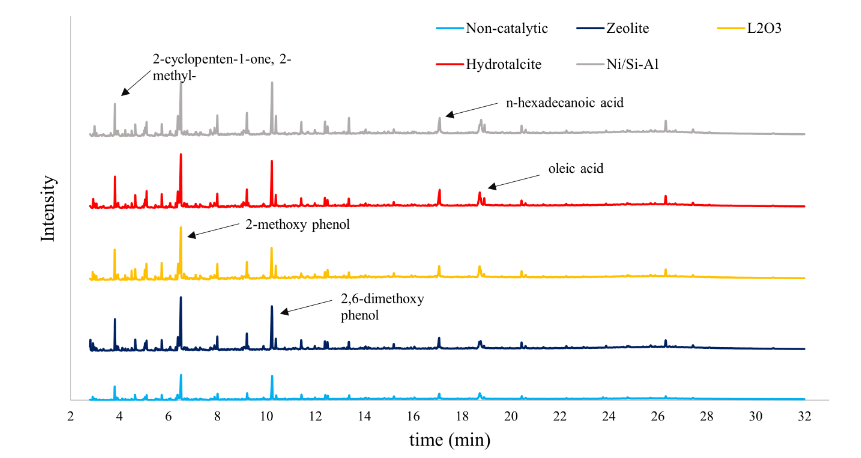


Figure S1. GC-MS peaks for each sample of the catalyst screening campaign.

The most prominent compounds that are noticed in every sample are phenols (2-methoxy and 2.6-dimethoxy phenols), alicyclic ketones (2-cyclopenten-1-one. 2-methyl-) and fatty carboxylic acids (n-hexadecanoic and oleic acids).

Table S7 - Table S11 show the GC-MS analysis of every compound detected in the bio-oil samples, along with the area percentage of each peak.

Table S7. GC- MS compounds of non-catalytic bio-oil sample.


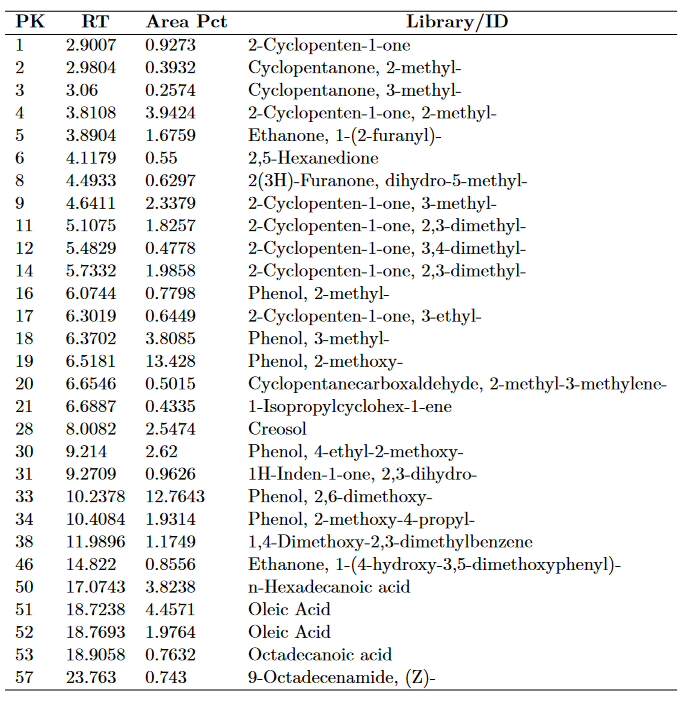


Table S8. GC- MS compounds of zeolite bio-oil sample.


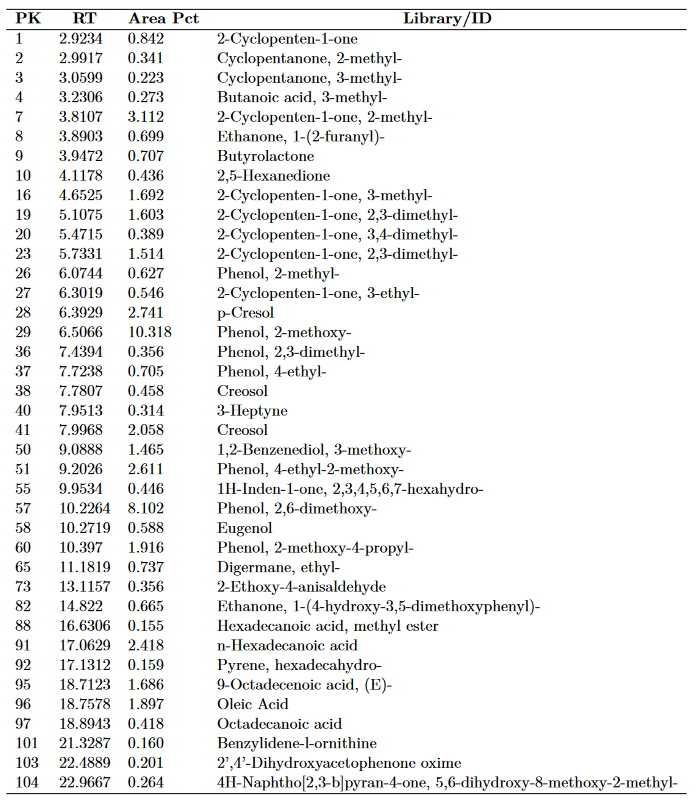


Table S9. GC- MS compounds of La₂O₃ bio-oil sample.


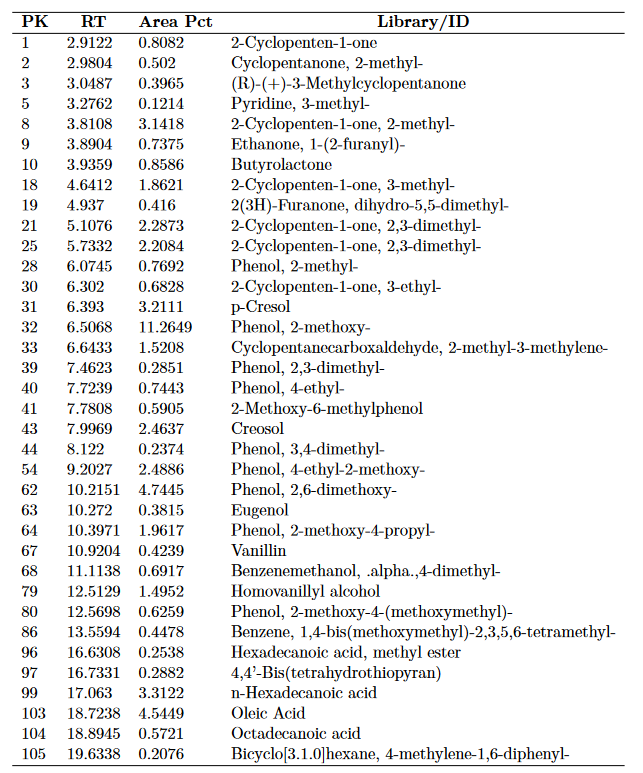


Table S10. GC- MS compounds of Hydrotalcite bio-oil sample.


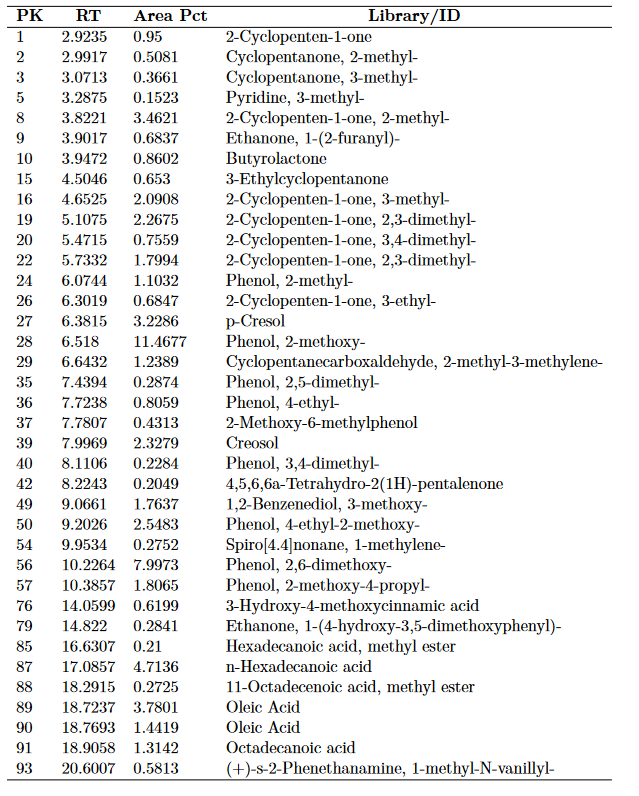


Table S11. GC- MS compounds of Ni/SiO₂-Al₂O₃ bio-oil sample.


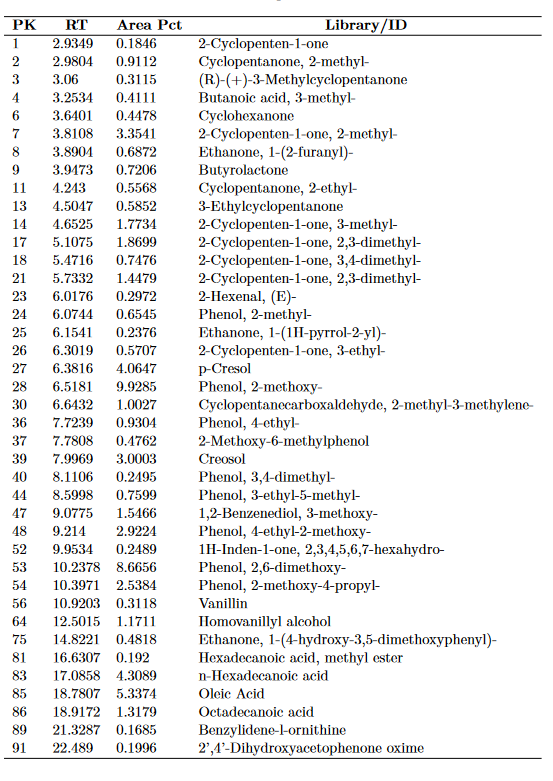


# CCD optimization campaign

Table S12 Randomized experimental matrix for CCD, provided by software Design Expert.


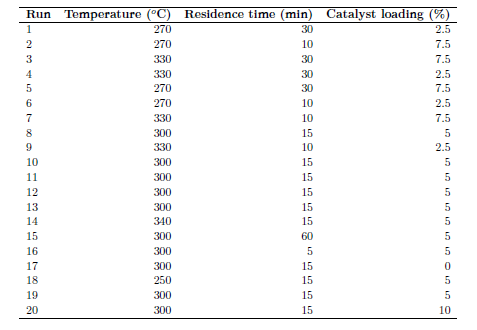


By entering the 3 different factors (temperature, residence time and catalyst loading) and their ranges, 20 different randomized combinations resulted, which were the 20 experiments performed.

Table S13 shows the predicted yields from the CCD for each product expressed in wt%.

Table S13. Predicted yields at validation point.


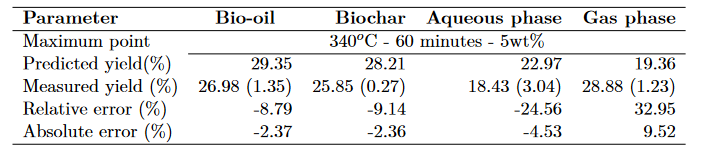


Bio-oil and biochar models had an overestimation in the model with a relative error similar to literature, while aqueous phase and gas phase models were underestimated and had a significant relative error.

Table S14 shows the coefficients for each quadratic equation of each HTL product.

Table S14. CCD quadratic model coefficients for each HTL product.


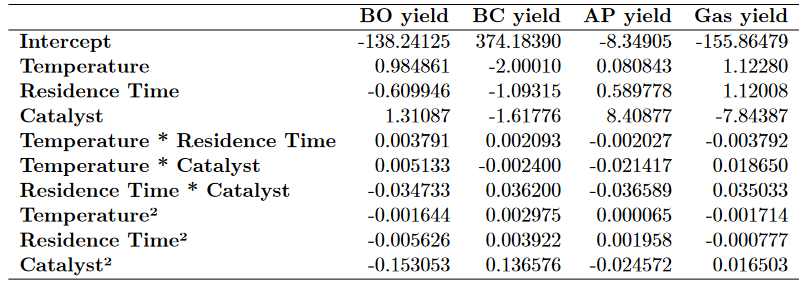


The quadratic equation format is the one below:

$$Yield\left( \% \right)=A_{0}+A_{1}*x+A_{2}*y+A_{3}*z+A_{4}*x*y+A_{5}*x*z+A_{6}*y*z+A_{7}*x^{2}+A_{8}*y^{2}+A_{9}*z^{2}$$

Where:

*x*: Temperature (^o^C), *y*: Residence time (min), *z*: Catalyst loading (wt%)

Table S15 presents the ultimate analysis for bio-oil and biochar from the CCD campaign.

Table S15. Ultimate analysis of bio-oils and biochar from CCD campaign

| Sample | C (%) | H(%) | N(%) | O(%) | H/C | O/C |
| --- | --- | --- | --- | --- | --- | --- |
| **Bio-oil** |  |  |  |  |  |  |
| BO-270-30-2.5 | 70.6 | 7.7 | 2.5 | 19.2 | 1.31 | 0.20 |
| BO-270-30-7.5 | 63.8 | 7.0 | 1.6 | 27.6 | 1.32 | 0.32 |
| BO-330-30-2.5 | 72.5 | 7.9 | 2.1 | 17.5 | 1.31 | 0.18 |
| BO-330-30-7.5 | 71.6 | 7.8 | 1.9 | 18.7 | 1.31 | 0.20 |
| BO-340-60-5 | 61.3 | 6.8 | 1.6 | 30.3 | 1.33 | 0.37 |
| **Biochar (d.b.)** |  |  |  |  |  |  |
| BC-330-30-7.5 | 58.7 | 5.0 | 2.2 | 34.1 | 1.02 | 0.44 |
| BC-330-30-2.5 | 69.3 | 5.3 | 2.6 | 22.8 | 0.92 | 0.25 |
| BC-270-30-2.5 | 64.9 | 5.7 | 3.1 | 26.3 | 1.05 | 0.30 |
| BC-270-30-7.5 | 59.3 | 5.5 | 2.2 | 33.0 | 1.11 | 0.42 |
| BC-340-60-5 | 63.2 | 4.9 | 2.2 | 29.7 | 0.93 | 0.35 |

It is observed that the samples 270-30-2.5, 330-30-2.5 and 330-30-7.5 have a similar distribution for C, H and O. In particular, the 330-30-2.5 sample had the highest C and H content of 72.5wt% and 7.9wt%, respectively. This sample also had the lowest O content at 17.5wt%. In comparison with the hydrotalcite sample from the catalyst screening campaign (at 300-15-5), the C, H and O content have all improved significantly, even for the low yield points at 270 ^o^C. The 340-60-5 point from the model had the lowest C content (61.3wt%) and highest O content (30.3wt%), making it the bio-oil with the lowest quality.

Regarding the biochar samples, the 330-30-2.5 one had the highest C content (69.3wt%) and lowest O content ( 22.8wt%). On the other hand, the biochar produced at 330-30-7.5 had the lowest C and O content at 58.7wt% and 34.1wt%, respectively.

Table S16 shows the proximate analysis of the biochar samples from the CCD campaign. The highest FC concentration was found in the BC-330-30-2.5 sample at 56.45wt%, while the least amount of VM was found in the BC-340-60-5 with 34.79wt%.

Table S16. Proximate analysis of CCD campaign for biochar samples (d.b.)

| Sample | Volatile Matter (VM) | Fixed Carbon (FC) | Ash |
| --- | --- | --- | --- |
| BC-330-30-7.5 | 35.40 ± 0.79 | 49.82 ± 1.09 | 14.78 ± 0.30 |
| BC-330-30-2.5 | 36.63 ± 1.45 | 56.45 ± 1.70 | 6.92 ± 0.25 |
| BC-270-30-2.5 | 46.03 ± 0.76 | 47.04 ± 0.89 | 6.93 ± 0.13 |
| BC-270-30-7.5 | 47.72 ± 0.77 | 43.12 ± 0.90 | 9.16 ± 0.13 |
| BC-340-60-5 | 34.79 ± 0.79 | 52.81 ± 1.94 | 12.40 ± 1.15 |

Table S17. HHV and bio-oil yields for the 5 CCD campaign points.

| Sample | HHV (MJ/kg) | HHV STD | Bio-oil yield |
| --- | --- | --- | --- |
| BO-270-30-2.5 | 27.20 | 1.19 | 19.43% |
| BO-270-30-7.5 | 25.65 | 1.02 | 18.96% |
| BO-330-30-2.5 | 30.04 | 0.30 | 25.60% |
| BO-330-30-7.5 | 31.34 | 0.22 | 28.48% |
| BO-340-60-5 | 28.34 | 0.36 | 26.97% |

Figure S2 presents the water content of the 5 CCD samples measured via Karl-Fischer Titration


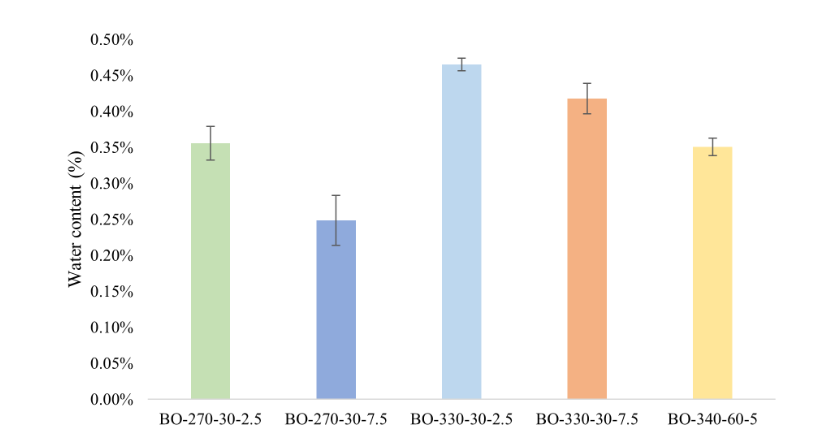


Figure S2. Water content of CCD bio-oil samples.

It is observed from Figure S2 that water content does not exceed 0.47wt% in any of the samples. The 270-30-7.5 point had the lowest water content with 0.25wt%. It appears that the samples with higher temperatures contain slightly more water than the ones with lower temperatures, but other than that, no discernible pattern exists between the samples.

Figure S3 shows the inorganic elemental composition of biochars from the CCD campaign.

**
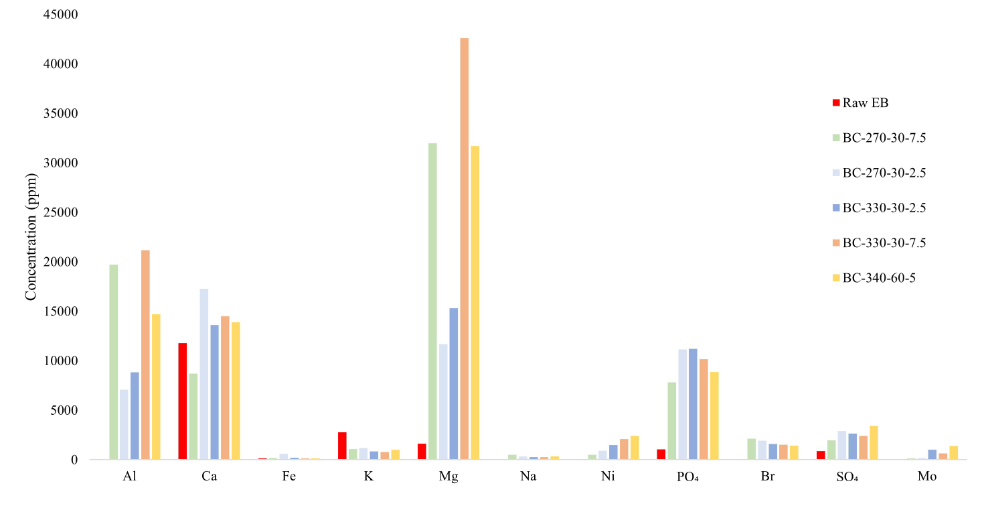
**

Figure S3. ICP-OES of biochars from the CCD campaign.

The concentration of Mg and Al was highest compared to other elements, mostly because they were present in hydrotalcite. This confirms the assumption that the catalyst deposits on the biochar. Furthermore, higher temperature samples seem to have higher Mg and Al concentrations. Moreover, heavy metals such as Ni and Mo are present in all the BC samples, whereas raw biomass did not have any. These elements came from the scraping of the inner autoclave’s walls with a metal spoon.

Figure S4 shows the distribution of inorganic elements in the aqueous phase of the non-catalytic and CCD campaign samples.

Figure S4. ICP-OES of the aqueous phase samples from the CCD campaign.

Al was introduced to the aqueous phase while using the hydrotalcite catalyst. Similarly, the presence of Mg was also greatly enhanced due to the composition of hydrotalcite, which mostly consists of Al and Mg. Moreover, Ni was reduced in all the catalytic samples compared to the non-catalytic one.

Table S18 - Table S22 show the compound distribution for the CCD campaign samples via the GC-MS analysis. The higher temperature samples (330 and 340 ^o^C) are less complex as these have much less compounds compared to the low temperature ones (270 ^o^C).

Table S18. GC-MS results for bio-oil 270-30-2.5


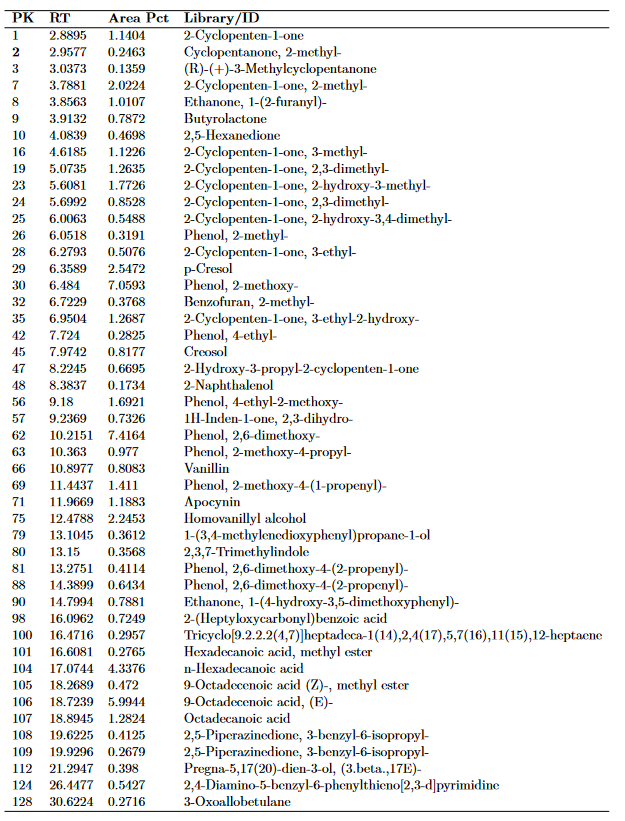


Table S19. GC-MS results for bio-oil 270-30-7.5


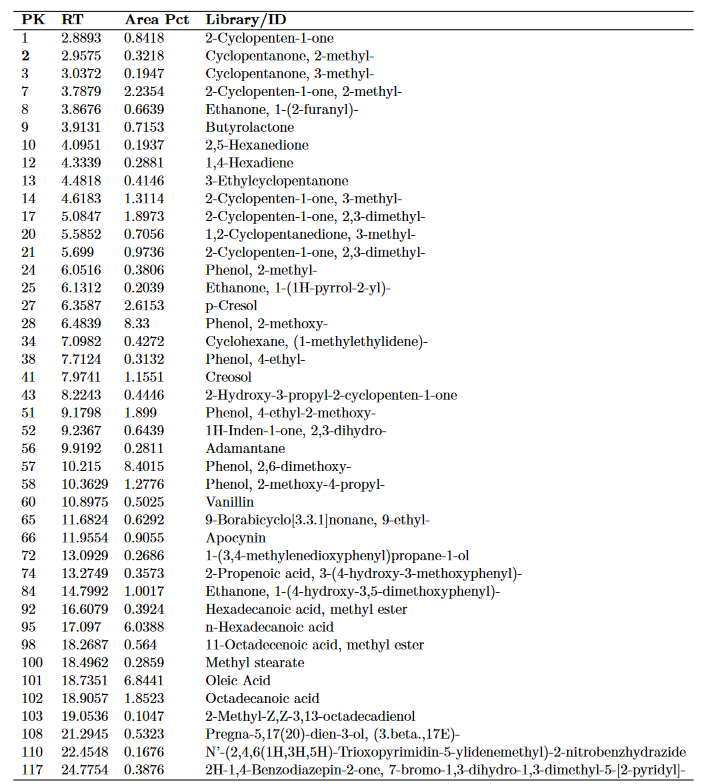


Table S20. GC-MS results for bio-oil 330-30-2.5


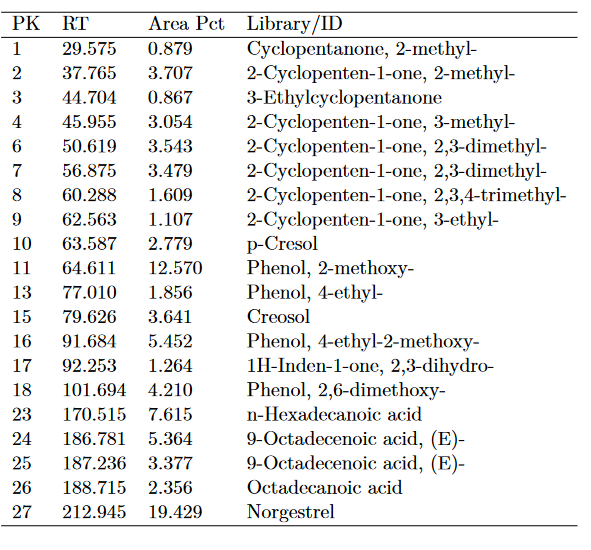


Table S21. GC-MS results for bio-oil 330-30-7.5


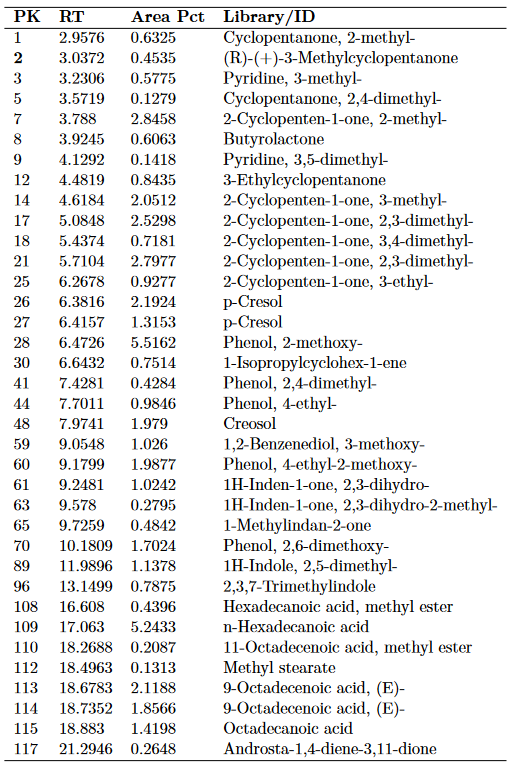


Table S22. GC-MS results for bio-oil 340-60-5


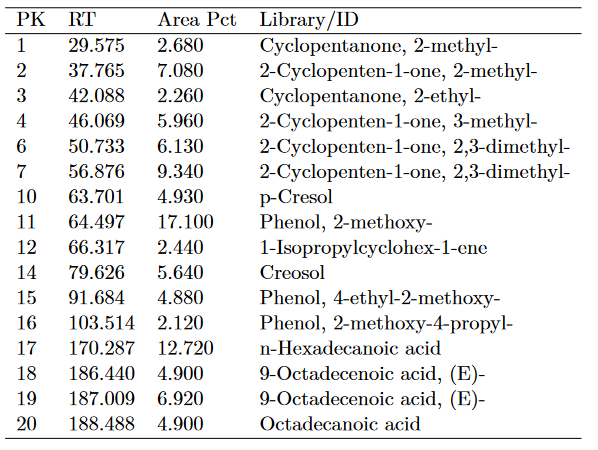


# Statistics for CCD optimization campaign

Table S23 to Table S26 show the ANOVA parameters from each phase predictive model. As discussed in the main text, the bio-oil, biochar and gas phase models are considered statistically significant, while the aqueous phase one is not.

Table S23. ANOVA parameters of the bio-oil yield model.


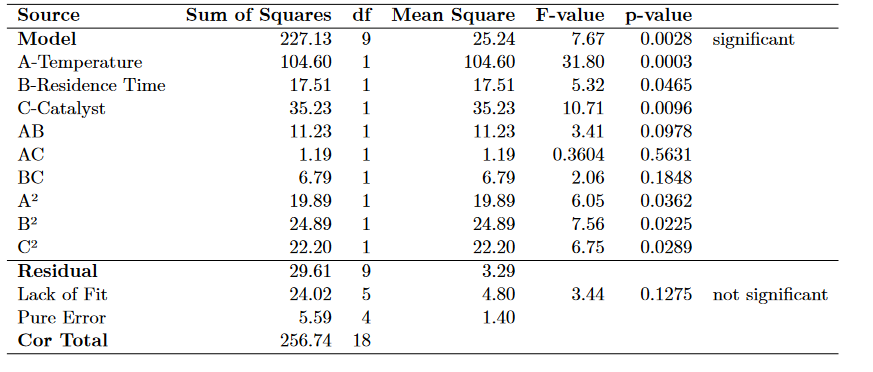


Table S24. ANOVA parameters of the biochar yield model.


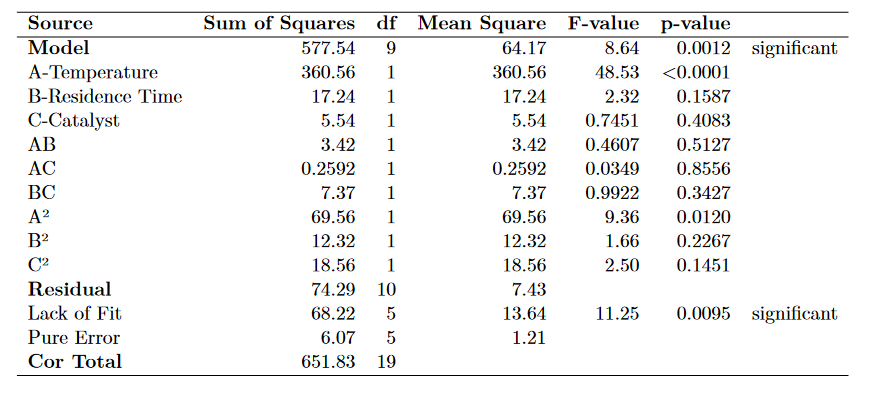


Table S25. ANOVA parameters of the aqueous phase yield model.


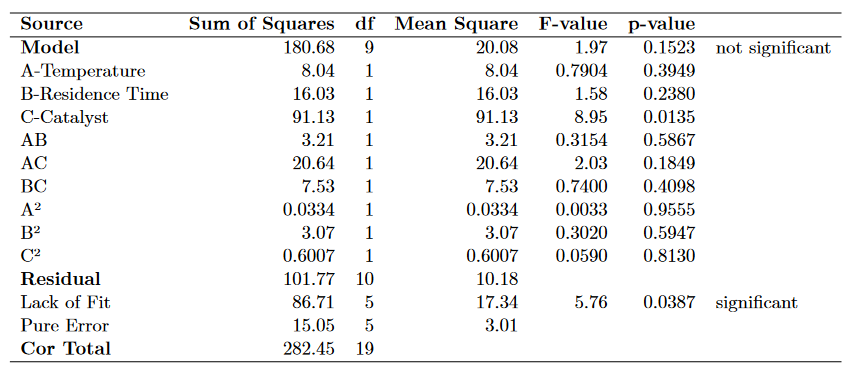


Table S26. ANOVA parameters of the gas phase yield model.


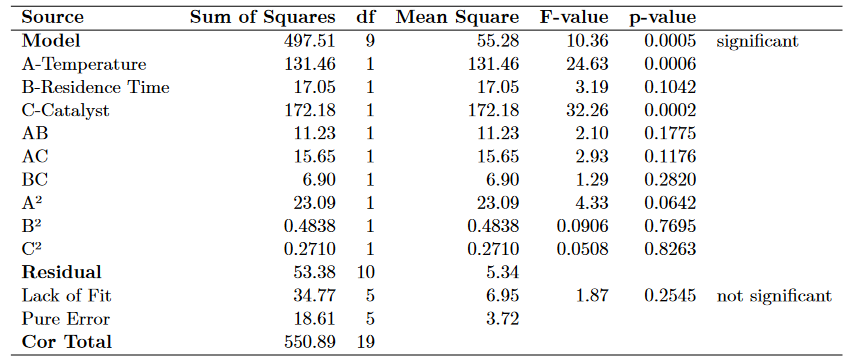


**References**

1. Reza, M. S. *et al.* Acacia Holosericea: An Invasive Species for Bio-char, Bio-oil, and Biogas Production. *Bioengineering* **6**, 33 (2019).

2. Reza, M. S. *et al.* Evaluation of the bioenergy potential of invasive Pennisetum purpureum through pyrolysis and thermogravimetric analysis. *Energ. Ecol. Environ.* **5**, 118–133 (2020).

3. Ahmed, A. *et al.* Characterization and Thermal Behavior Study of Biomass from Invasive Acacia mangium Species in Brunei Preceding Thermochemical Conversion. *Sustainability* **13**, 5249 (2021).
